# Development of Silica Nanoparticles Embedded Adipose Spheroid Platform for Probing Bacteriophage Sequestration and Its Implications for Phage Therapy

**DOI:** 10.3390/nano15191537

**Published:** 2025-10-09

**Authors:** Rafael Levandowski, Su Yati Htun, Laura Ha

**Affiliations:** Department of Pharmaceutical Engineering and Biotechnology, Sun Moon University, 70 Sun Moon-ro 221, Tangjeong-Myeon, Asan-si 31460, Chungnam, Republic of Korea; rafaelldk28@gmail.com (R.L.); suya1112@sunmoon.ac.kr (S.Y.H.)

**Keywords:** human adipose-derived stem cells, 3D spheroids, mesoporous silica nanoparticles, adipogenesis, bacteriophage T4

## Abstract

We engineer an enhanced three-dimensional (3D) adipose model by integrating mesoporous silica (mSiO_2_) nanoparticles into human adipose-derived stem cell spheroids. The mSiO_2_ is highly cytocompatible, enables stable dispersion, and yields spheroids that preserve structural integrity and roundness for at least 14 days, accompanied by higher metabolic activity and reduced hypoxic stress. Under adipogenic induction, the nanoparticles embedded spheroids exhibit deeper lipid accumulation and increased expression of PPARγ, adiponectin, and FABP4. As a proof of concept, we leveraged this 3D platform to examine phage uptake and tissue-level distribution in adipose spheroids in comparison with conventional 2D cultures. These experiments reveal that both the cellular differentiation state and the tissue architecture govern phage association and uptake. Together, our findings indicate that phages engage mammalian cells beyond their bacterial hosts, a consideration that should inform future phage therapy design with implications for innate immune responses and overall therapeutic efficacy.

## 1. Introduction

Two-dimensional (2D) cell culture models often fail to recapitulate the physiological behavior of cells observed in living tissues, as cells cultured on flat surfaces may develop unnatural polarity and morphology [1]. This can lead to altered signaling and function compared to cells growing in their native biological environment. Three-dimensional (3D) culture systems, such as spheroids and organoids, have thus been developed to better mimic native microenvironments, as these platforms more effectively induce in vivo-like cell fates and responses [2,3,4]. These advantages have enabled the application of 3D culture systems across various research and clinical fields, including cancer biology, stem cell research, drug screening, and tissue engineering. As 3D culture technologies continue to evolve, they are expected to bridge the gap between in vitro experimentation and in vivo conditions, ultimately accelerating translational research and the development of more effective therapeutic interventions [5].

Despite their advantages, 3D culture systems such as spheroids and organoids often develop a necrotic core due to limited oxygen and nutrient diffusion, particularly in larger constructs. This hypoxic and nutrient-deprived center can lead to unintended cell death and altered cellular behavior, which may compromise the physiological relevance and reproducibility of experimental results [6].

**Figure 1:**
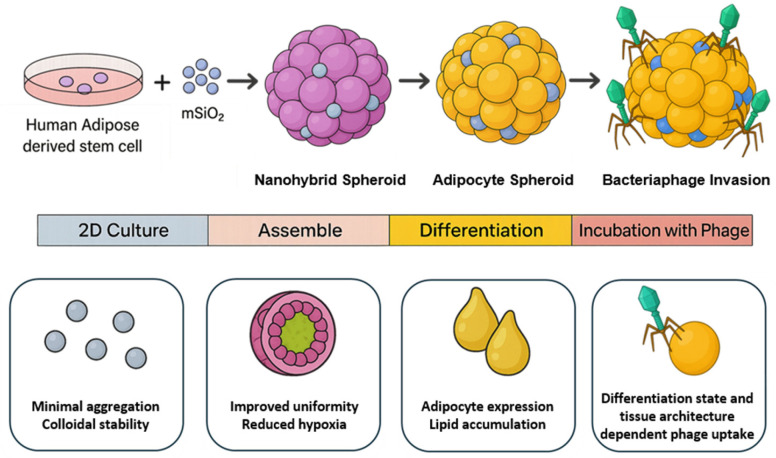
Schematic workflow for the generation and application of nanohybrid adipose spheroids.

To address the challenge of necrotic core formation in conventional 3D spheroid models, here, we propose a novel strategy that incorporates mesoporous silica nanoparticles (mSiO_2_) into adipose spheroids (Figure 1); in this study, we focus on adipose spheroids as a representative model system due to the metabolic complexity and physiological relevance of adipose tissue. We selected mSiO_2_ because of its excellent biocompatibility, high surface area, and well-defined porosity, which make it an ideal filler for 3D spheroid systems [7]. These properties enable both efficient loading of bioactive agents and improved intra-spheroidal transport of oxygen and nutrients, thereby mitigating hypoxic core formation and supporting long-term metabolic stability [7,8,9,10,11,12,13]. At the same time, mSiO_2_ incorporation provides mechanical reinforcement—enhancing structural strength so that spheroids can preserve their three-dimensional architecture and resist deformation during culture [8,9]. As a result, the spheroid microenvironment is supplemented not only with biochemical and mechanical support but also with a sustained local supply of metabolic substrates, ultimately creating a more physiologically relevant and functionally robust adipose tissue model.

Building on this platform, we further explored its utility to probe bacteriophage–mammalian cell interactions under tissue-like constraints. Interest in phage therapy for drug-resistant infections is rising; however, in vivo efficacy remains limited by short intrabody half-lives and host clearance, including adherence to and uptake by mammalian cell layers that act as phage sinks [14,15,16,17,18,19]. Because 2D cultures do not capture diffusion barriers or cell–cell/ECM contacts, our 3D model enables spatiotemporal mapping of phage access, surface association, and uptake across pre-adipose and adipocyte states. These studies provide insight into phage distribution in adipose tissue and establish a physiologically relevant platform for evaluating tissue-targeted phage strategies.

## 2. Materials and Methods

### 2.1. Chemicals and Materials

Tetraethyl orthosilicate (TEOS, ≥99%), ammonium hydroxide solution (28% *w*/*w*), absolute ethanol (≥99.5%), hexadecyltrimethylammonium bromide (CTAB, ≥99%), and 3-aminopropyltriethoxysilane (APTES, ≥98%) were purchased from Sigma-Aldrich (St. Louis, MO, USA). Human adipose-derived stem cells (hADSCs), StemPro™ Adipose-Derived Stem Cell Medium, Dulbecco’s phosphate-buffered saline (DPBS), trypsin-EDTA, penicillin–streptomycin, and Hoechst 33342 solution (20 mM) were obtained from Thermo Fisher Scientific (Waltham, MA, USA). Ultra-low attachment (ULA) 96-well plates were purchased from Corning Incorporated (Corning, NY, USA). CellTiter-Glo^®^ 3D Cell Viability Assay kits were obtained from Promega (Madison, WI, USA), and CCK-8 kits were purchased from Dojindo Laboratories (Kumamoto, Japan). The LOX-1 fluorescent hypoxia probe was acquired from Abcam (Cambridge, UK). All other reagents and solvents were of analytical grade and used as received.

### 2.2. Synthesis and Characterization of Mesoporous Silica Nanoparticles

The mSiO_2_ nanoparticles were synthesized via previously reported method [20,21]. Briefly, CTAB (0.29 g) was dissolved in 150 mL of 0.51 M ammonium hydroxide under stirring (500 rpm) at 50 °C for 1 h. A silica precursor solution (3 mL; 0.88 M TEOS in ethanol with 1.5 µL APTES) was added dropwise and stirred for an additional hour. Note that APTES functionalization was employed to introduce surface amine groups on MSNs, which enhance interactions with cells and colloidal stability in aqueous media [22,23]. The mixture was aged statically at 50 °C for 18 h, filtered (1.0 µm), and hydrothermally treated at 70 °C for 24 h. Template removal involved refluxing in 75 mM ammonium nitrate solution in ethanol (60 °C, 1 h) and acidic washing in 12 mM HCl ethanol solution (60 °C, 2 h). Nanoparticles were centrifuged (10,000× *g*, 15 min) and dried in oven. Morphology was analyzed via SEM (Hitachi SU3500, Hitachi High-Technologies, Tokyo, Japan). Hydrodynamic diameter and PDI were measured by dynamic light scattering (DLS) in PBS (Zetasizer Nano ZS, Malvern Panalytical, Malvern, UK). Zeta potential was assessed across ionic strengths (0–0.5 M NaCl) and pH (2–10). Thermogravimetric analysis (TGA) was performed to confirm amine group functionalization on mSiO_2._

### 2.3. Spheroid Formation and Functional Assessment

hADSCs were maintained in StemPro™ culture medium supplemented with 1% penicillin–streptomycin under standard culture conditions (37 °C, 5% CO_2_). For spheroid formation, 10,000 cells were resuspended in fresh medium containing either 0 (control), 100, or 200 ng mSiO_2_ preloaded with essential nutrients and adipogenic agents and seeded into each well of a 96-well ULA round-bottom plate in 200 µL of total volume. Then, the plate was centrifuged at 1500 rpm for 1 min to initiate cell aggregation and then incubated statically for up to 14 days. Half of the medium was refreshed after 24 h and afterwards every 2–3 days.

### 2.4. Morphological and Viability Assessment of 3D Spheroids

Spheroid morphology was monitored daily using phase-contrast microscopy (Nikon Eclipse Ti, Nikon Instruments Inc., Tokyo, Japan). ImageJ (version 1.54p, National Institutes of Health, Bethesda, MD, USA) was used to quantify diameter and circularity (defined as C = $\frac{4\pi \;x\;area}{{\mathrm{perimeter}}^{2}}$) from bright-field images (*n* = 10 spheroids per group) [24]. At selected time points (days 1, 7, and 14), intracellular ATP content was measured using the CellTiter-Glo^®^ 3D assay, in a Synergy H1 plate reader (BioTek Instruments, Winooski, VT, USA). Hypoxia within the spheroids was assessed using a live-cell fluorescent LOX-1 probe (4 µM final concentration). Spheroids were incubated overnight with the probe, washed in PBS, and transferred to black-walled 96-well plates. Fluorescence intensity (λ_ex_ 484 nm/λ_em_ 616 nm) was recorded using the same plate reader and normalized to background signal. For SEM imaging, spheroids were fixed in 4% paraformaldehyde, dehydrated in graded ethanol, dried by critical point drying, and sputter-coated with gold. Surface architecture was examined using SEM at 10 kV. Additionally, fixed spheroids were embedded in paraffin, sectioned, and stained with hematoxylin and eosin (H&E) for histological evaluation under bright-field microscopy.

### 2.5. Adipogenic Differentiation of 3D Spheroids and Genetic Analysis

To induce adipogenic differentiation, the growth medium was changed to adipogenic differentiation media containing 10% FBS, 1% P/S, 0.5 mM 3-isobutyl-1-methylxanthine, 0.2 mM indomethacin, 1 μg/mL insulin, and 1 μM dexamethasone for 7 days. Spheroids cultured in growth media were used as control. At the end of 7 days differentiation period, total RNA was extracted from spheroids using a standard phenol–chloroform protocol (TRIzol). RNA concentration and purity were determined spectrophotometrically (NanoDrop, Thermo Fisher Scientific, USA). Complementary DNA (cDNA) synthesis was performed with AccuPower^®^ RT PreMix (Bioneer Inc., Daejeon, Republic of Korea) using reverse transcriptase and oligo(dT) primers according to the manufacturer’s instructions. RT-PCR amplification was carried out for the following adipogenic markers: 18S ribosomal RNA (housekeeping gene), PPARγ, adiponectin, and FABP4 (Figure S3). For each PCR reaction, 10–50 ng of cDNA template was used, and the same concentration was applied for both target genes and the 18S rRNA housekeeping control to enable reliable normalization. For agarose gel electrophoresis, 10–12 µL of each PCR product was loaded per lane along with a DNA size marker (100–3000 bp DNA ladder, Thermo Fisher Scientific, USA). Band intensities of target genes were quantified by densitometry and normalized against the corresponding 18S rRNA band intensity. PCR products were separated by agarose gel electrophoresis (2%), stained with SYBR Safe, and visualized under UV illumination. Gene expression levels were estimated semi-quantitatively via densitometric analysis of agarose gel bands using ImageJ software (NIH, Bethesda, MD, USA), normalized to 18S.

### 2.6. Histological and Lipid Accumulation Analysis of Spheroids

For histological assessment, spheroids were fixed in 4% paraformaldehyde, embedded in paraffin, and sectioned at 5 µm thickness. Sections were stained with H&E to evaluate cell and tissue morphology following differentiation. To assess lipid accumulation, spheroid sections were stained with lipid (Oil Red O) staining kit (Sigma, USA). Briefly, paraffin sections were deparaffinized, rehydrated through graded alcohols, and incubated in Oil Red O working solution for 15 min. Sections were counterstained with hematoxylin and mounted with aqueous mounting medium. Stained lipid droplets were visualized by bright-field microscopy, and positively stained areas were quantified using ImageJ. The lipid-positive area was expressed as a percentage of the total spheroid section area, and data were presented graphically for each experimental group.

### 2.7. Bacterial Host and Bacteriophage T4 Propagation

*Escherichia coli* BL21(DE3) (*E. coli*) was utilized as the bacterial host strain for bacteriophage T4 propagation. Initially, *E. coli* was cultured in LB broth (Invitrogen, Carlsbad, CA, USA) at 37 °C under shaking conditions (160–180 rpm). Cultivation continued until the culture reached an optical density at 600 nm (OD600) of 0.1–0.3, measured using a spectrophotometer. Bacteriophage T4 (ATCC, Manassas, VA, USA) was purchased and initially thawed gently on ice. To propagate the bacteriophage, a 5 mL aliquot of bacterial culture at the appropriate OD600 was infected with 100 µL of the T4 stock. The infection mixture was incubated overnight at 37 °C under static conditions. Following propagation, the bacteriophage-containing lysate was centrifuged at 4000× *g* for 10 min to sediment bacterial debris. The resulting supernatant was filtered through a sterile 0.22 µm polyethersulfone (PES) membrane (MilliporeSigma Burlington, MA, USA) to obtain purified bacteriophage lysates. The bacteriophage titer was determined by a standard spot titer assay using soft agar overlays (0.5% agar in LB), adjusted to approximately 10^7^ PFU/mL, and stored at 4 °C in sterile SM buffer (Tecnova Inc., Hollister, CA, USA) until use [25].

### 2.8. T4 Bacteriophage Penetration Assay

For penetration assays, 50 µL of the bacteriophage working solution was gently added to each well containing spheroids, bringing the final assay volume to 100 µL per well [23]. The mixture was incubated at 37 °C in a humidified atmosphere containing 5% CO_2_ for 2 h without agitation to facilitate bacteriophage–spheroid interactions. After incubation, spheroids were gently but thoroughly washed three times with sterile PBS to remove loosely bound or non-internalized bacteriophages. Washing steps involved careful aspiration and slow addition of PBS to minimize spheroid disruption. Spheroids were then stained with SYBR^®^ Gold nucleic acid stain (Biotium, Fremont, CA, USA) to label bacteriophage and with Hoechst 33342 (20 µM; Thermo Fisher Scientific, USA) to counterstain cell nuclei. A fresh 1:10,000 dilution of SYBR Gold and the Hoechst solution were prepared in sterile PBS, and 100 µL of the combined staining solution was added per well. Spheroids were incubated for 15 min at room temperature in the dark, followed by an additional PBS washing step to eliminate excess dye and reduce background fluorescence. Fluorescence microscopy was performed using an inverted fluorescence microscope (Olympus Corporation, Tokyo, Japan). Images were captured under consistent microscope settings, utilizing excitation at approximately 495 nm and emission at approximately 537 nm. Both brightfield and fluorescence images were collected, and Z-stack imaging was conducted at intervals of 5–10 µm through the entire spheroid depth. Images were saved in lossless TIFF format for subsequent analysis.

### 2.9. Statistical Analysis

All quantitative data are presented as mean ± standard deviation (SD) unless otherwise stated. Statistical analyses and graphic drawings were performed using GraphPad Prism software (version 10.0; GraphPad Software, San Diego, CA, USA). One-way analysis of variance (ANOVA) was used to compare means between groups, followed by Tukey’s post hoc test for multiple comparisons. A *p*-value < 0.05 was considered statistically significant.

## 3. Results

### 3.1. Characterization of mSiO_2_ Nanoparticles

As shown in Figure S1A, the synthesized mSiO_2_ nanoparticles exhibited a spherical shape. Surface charge measurements demonstrated a pronounced modulation of zeta potential in response to ionic strength and pH variations (Figure S1B,C). At physiological conditions, the nanoparticles exhibited a negative surface potential (~−25 mV), indicative of robust colloidal stability due to electrostatic repulsion. Particle size analysis confirmed consistent nanoparticle synthesis, with an average diameter of 119.8 nm (Figure S1D). The thermogravimetric analysis (TGA) for amine functionalized mSiO_2_ nanoparticles has been performed (Figure S1E). Consistent with previous reports, distinct weight loss steps were observed in the TGA curves [26]. The first step at temperatures 100 °C is due to the removal of physically absorbed water and ethanol. At 230 °C indicates the removal of volatile silanols and surface hydroxyl groups at the surface of silica nanoparticles, beyond which, the weight loss is owned to the physical adsorption of silane coupling agents. Decomposition of the amine functional groups attached by strong chemical bonds is responsible for the second weight loss, in the range between 500 and 600 °C, which accounts for about 6% of the total weight loss [21,26].

Cytocompatibility assessments using NIH 3T3 fibroblasts indicated excellent biocompatibility at both nanoparticle concentrations (4 and 40 µg/mL), with viability consistently above 95% up to 7 days (Figure S1E). These results support previous findings that mSiO_2_ nanoparticles exhibit inherently low toxicity due to rapid dissolution into non-toxic silicic acid species and reduced cell membrane interaction due to their mesoporous structure. Altogether, we confirmed that these mSiO_2_ particles as a suitable candidate for incorporation into hADSC-based spheroid models.

### 3.2. Structural and Functional Evaluation of hADSC Spheroids Formed with Different Concentrations of mSiO_2_

**Figure 2:**
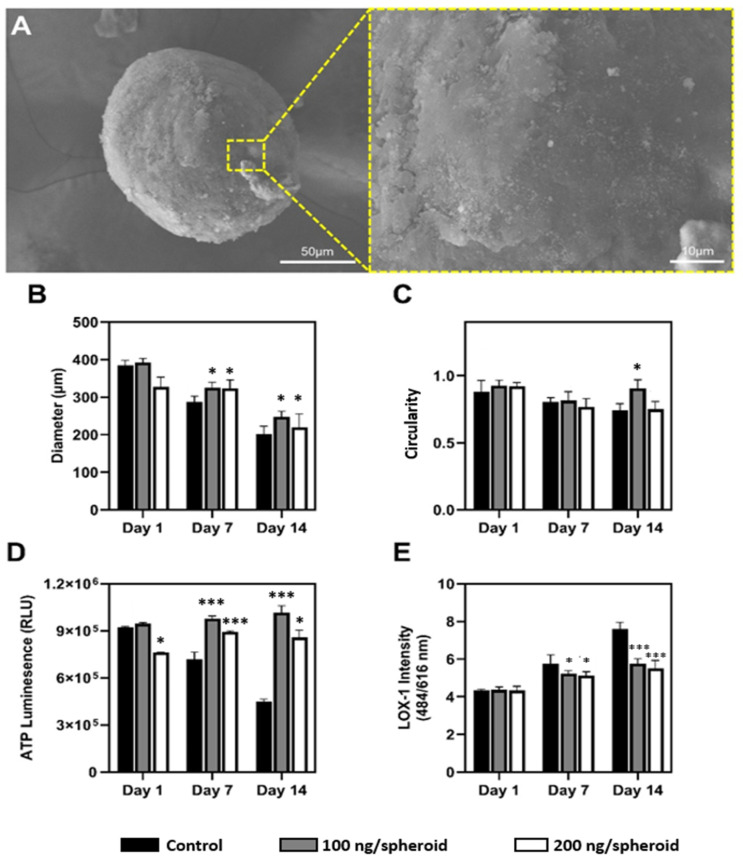
Morphological and metabolic characterization of 3D hADSC spheroids formed with different concentrations of mSiO_2_. (**A**) Scanning electron microscopy (SEM) image of a representative spheroid containing mSiO_2_ at day 7. (**B**) Spheroid diameter over 14 days in the presence of 0 (black), 100 (gray), and 200 ng/spheroid (white) mSiO_2_ (*n* = 10/group). (**C**) Spheroid circularity across time points and treatment groups (*n* = 10/group). (**D**) Intracellular ATP content (*n* = 10/group). (**E**) LOX-1 fluorescence intensity (λ_ex 484 nm/λ_em 616 nm) (*n* = 10/group). * *p* < 0.05, *** *p* < 0.005 vs. control spheroid in same day.

Nanoparticles embedded spheroids (nanohybrid spheroids) were created by seeding 10,000 hADSCs with mSiO_2_ (0, 100 ng, or 200 ng) into ULA plates, followed by centrifugation and static culture for up to 14 days. Shown in Figure 2A and Figure S2A, the nanohybrid spheroids demonstrated consistent structural integrity up to 14-day cultivation period, as evidenced by SEM image; the compact and continuous surface topology indicates mechanical stability and integrity over prolonged culture durations.

Nanoparticle concentration-dependent influence on spheroid diameter and circularity were analyzed quantitatively over 14 days. As shown in Figure 2B, initially, all spheroids presented comparable diameters. However, nanoparticle incorporation affected both the rate and extent of size reduction. Control spheroids showed the greatest diameter reduction by day 14 while spheroids treated with 100 ng/spheroid nanoparticles exhibited the most gradual and controlled compaction (−36.9%). Similar results were observed in spheroid circularity (Figure 2C). Initially, all groups exhibited high sphericity, but by day 14, the control group showed significant morphological distortion, with circularity dropping below 0.75, while the 100 ng/spheroid condition consistently maintained high circularity (>0.90). These results suggest that the presence of nanoparticles enhances structural uniformity and spatial consistency, both of which are critical for uniform nutrient diffusion and metabolic function [27,28,29].

Next, metabolic variations between nanoparticle-treated and untreated groups were assessed via ATP quantification (Figure 2D). Control spheroids showed a decline in metabolic activity over time, likely due to limited nutrient diffusion and increased hypoxic stress typical of dense spheroid structures [30]. In contrast, spheroids treated with 100 ng/spheroid nanoparticles exhibited the highest ATP levels by day 14, surpassing initial values—indicating enhanced cell viability and sustained metabolic function. These findings suggest that nanoparticle incorporation improves nutrient transport and reduces hypoxia, thereby supporting prolonged cellular functionality [9].

Furthermore, hypoxia-associated stress was monitored via LOX-1 fluorescence (Figure 2E). A progressive increase in hypoxic stress was observed across all groups, but with notable differences in magnitude. Control spheroids exhibited sharply rising LOX-1 signals, reflecting limited oxygen and nutrient diffusion typical of dense spheroid structures [31]. In contrast, nanohybrid spheroids showed attenuated hypoxic responses, with the 100 ng/spheroid consistently exhibiting the lowest fluorescence intensity. These findings suggest that mSiO_2_ nanoparticles enhance intra-spheroidal diffusion, mitigating core hypoxia and related cellular stress, consistent with previous reports on improved mass transport in nanoparticle-integrated systems.

**Figure 3:**
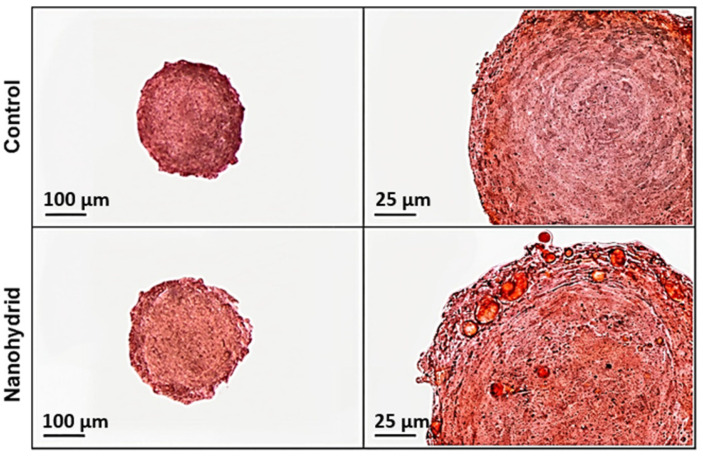
Histological evaluation of adipogenic differentiation in control and nanohybrid adipose-derived spheroids using Oil Red O staining.

These findings were further supported by H&E (Figure S2B). H&E staining revealed a heterogeneous and disorganized internal architecture in the control group; the central region, outlined by the dashed box, exhibited features indicative of core hypoxia, including reduced cell density and disrupted extracellular matrix integrity (Figure 3). This disorganized morphology aligns with the elevated LOX-1 fluorescence (Figure 2E) and the significant loss of spheroid circularity, both of which are indicative of progressive mechanical instability and impaired nutrient and oxygen diffusion within the control spheroids [32]. In contrast, nanohybrid spheroids exhibited significantly improved structural organization. Although both groups exhibited a gradual reduction in spheroid diameter by day 14, circularity values remained relatively stable, likely because compaction occurred uniformly rather than asymmetrically. This more homogeneous architecture, together with improved nutrient and oxygen diffusion, may explain why spheroids became smaller yet retained spherical morphology throughout culture [9]. Their overall morphology appeared more uniform and cohesive, with no evident hypoxic cores or major architectural disruptions; cellular distribution remained homogeneous throughout the spheroid. It is worth noting that previous studies have highlighted the critical role of mechanical support in spheroids for maintaining cellular viability and preventing necrosis [7,8]. Based on these findings, it is plausible that the enhanced structural integrity observed in our nanohybrid spheroids contributed to the improved metabolic performance, as seen in the higher ATP content (Figure 2D) and reduced hypoxic stress in the nanohybrid group.

### 3.3. Analysis of Adipose-Derived Spheroids

The structural and functional evaluations demonstrate concentration-dependent effects of mSiO_2_ nanoparticles. Based on these results, the 100 ng/spheroid concentration was selected, as it effectively balanced structural integrity, morphological uniformity, metabolic viability, and reduced hypoxic stress. To develop adipose-derived spheroids, the growth medium was replaced with adipogenic differentiation media for 7 days.

Firstly, lipid accumulation was analyzed via Oil Red O staining. Shown in Figure 3, pronounced adipogenic differentiation in both control and nanohybrid adipose-derived spheroids, with morphological differences in lipid accumulation. Control spheroids exhibited lipid droplets that were generally smaller and often confined towards the spheroid periphery, suggesting a more heterogeneous or regionally limited differentiation. In contrast, nanohybrid spheroids showed a more homogeneous distribution of Oil Red O–positive lipid droplets across the 3D structure. While lipid accumulation was not fully uniform throughout the spheroid, lipid droplets extended deeper toward the core compared to the control group, indicating enhanced and spatially expanded adipogenic differentiation. Indeed, large unilocular droplet formation within the nanohybrid spheroids provides morphological evidence of more advanced adipogenic maturation, closely mimicking native adipose tissue architecture. This enhanced lipid filling throughout the nanohybrid indicates that the inclusion of mSiO_2_ not only increases the extent of differentiation but also promotes a spatially uniform maturation of adipocytes within the spheroid volume [7,8,9].

**Figure 4:**
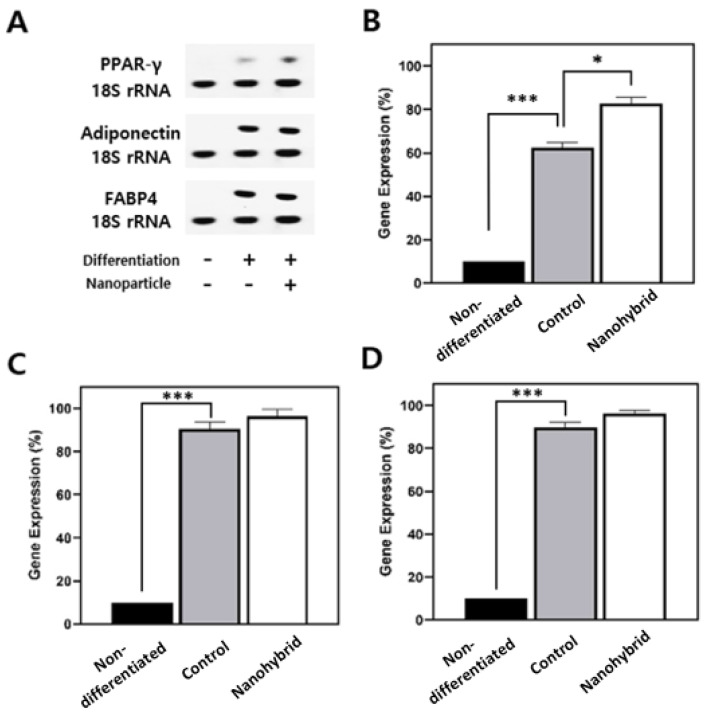
Adipogenic gene expression in hADSC-derived spheroids. (**A**) Representative endpoint RT-PCR/gel images for PPARγ, adiponectin, and FABP4 with 18S rRNA as an internal control. Lanes correspond to non-differentiated (−/−), differentiated control (+/−), and differentiated nanohybrid with mSiO_2_ (+/+). Quantitative gene expression (%), normalized to 18S rRNA, (**B**) for PPARγ, (**C**) for adiponectin, and (**D**) for FABP4. (*n* = 3/group). * *p* < 0.05, *** *p* < 0.005.

Next, molecular analysis of adipogenic differentiation markers was performed via PCR after RNA extraction and cDNA synthesis (Figure 4). Compared to controls, nanohybrid spheroids showed significantly higher expression of key adipogenic genes—PPARγ, adiponectin, and FABP4—indicating a more robust activation of the adipogenic program.

PPARγ, a master regulator of adipogenesis, was markedly upregulated, suggesting strong adipogenic commitment and supporting the increased lipid accumulation observed histologically. Adiponectin, a marker of functional maturity in adipocytes, was also elevated, reflecting enhanced endocrine functionality. Additionally, FABP4, involved in intracellular lipid handling, was robustly induced, consistent with the presence of larger lipid droplets.

The concurrent upregulation of these markers confirms that nanohybrid spheroids underwent more complete and functional adipogenic differentiation, both morphologically and molecularly. Together, we could confirm that nanohybrid spheroids closely mimic native adipose tissue in both structure and function, highlighting the role of mesoporous silica nanoparticles in supporting robust adipogenic differentiation and tissue-like maturation [9,10,20].

### 3.4. Modeling Bacteriophage Uptake and Tissue-Level Distribution in Adipose Spheroids

**Figure 5:**
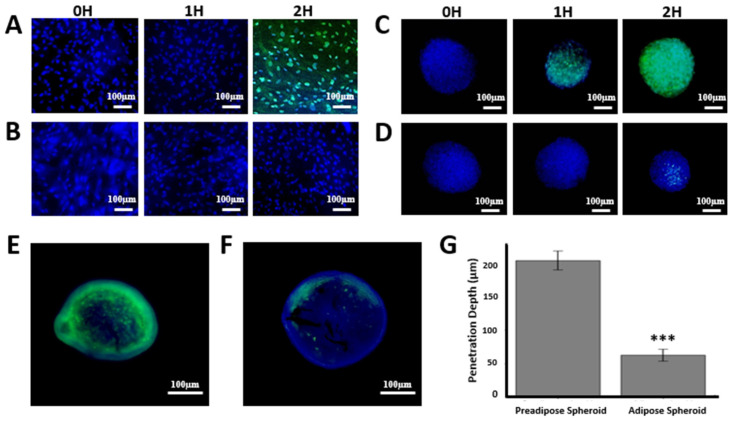
Architecture- and maturation-dependent uptake of T4 in hADSC-derived adipose models. (**A**) Undifferentiated pre-adipose cells and (**B**) Differentiated adipose cells in 2D monolayers imaged at 0, 1, and 2 h after phage exposure. (**C**) Pre-adipose 3D spheroids and (**D**) Adipose 3D spheroids at 0, 1, and 2 h. Fluorescence image of a cryosectioned (**E**) Pre-adipose 3D spheroid and (**F**) adipose 3D spheroid 2 h after exposure to T4 bacteriophages. Scale bars: 100 μm. DAPI (blue) labels nuclei; SYBR Gold–labeled bacteriophage T4 appears green. (**G**) Average penetration depth of bacteriophages into pre-adipose and adipose 3D spheroids (*n* = 5), *** *p* < 0.005 vs. pre-adipose spheroid.

As a proof of concept, we applied the adipose nanohybrid spheroid model to examine bacteriophage–host interactions under tissue-like constraints. In 2D cultures, SYBR Gold–labeled T4 phages readily entered pre-adipose cells, with minimal signal at 0–1 h and robust cytoplasmic accumulation by 2 h (Figure 5A). In contrast, differentiated adipocytes displayed no detectable uptake within the same timeframe (Figure 5B), indicating markedly reduced susceptibility.

In 3D nanohybrid spheroids, pre-adipose surfaces showed gradual accumulation beginning at 1 h and widespread penetration by 2 h (Figure 5C). Adipose spheroids, however, exhibited delayed and heterogeneous signal, first appearing at 2 h in a patchy distribution (Figure 5D). This delay was more pronounced than in corresponding 2D adipose cultures.

Cryosectioning and fluorescence imaging at 2 h revealed stark differences in penetration depth. Pre-adipose spheroids showed strong phage fluorescence extending deep into the core (203.9 ± 14.1 µm), whereas adipose spheroids exhibited signal largely confined to the periphery (62.9 ± 9.0 µm) (Figure 5E–G).

Together, these results demonstrate that both cellular differentiation state and tissue architecture strongly shape the penetration and distribution dynamics of phages within 3D adipose spheroids. It should be emphasized that these observations reflect non-specific adherence, penetration, and transient uptake of native T4 phages, rather than productive infection of mammalian cells.

## 4. Discussion

The integration of mSiO_2_ into spheroids significantly influenced their structural integrity, addressing one of the primary limitations of conventional 3D culture systems. In typical spheroid models, necrotic cores often develop due to limited oxygen and nutrient diffusion, which can compromise viability and alter cell behavior. In our study, control spheroids displayed progressive diameter reduction, loss of circularity, and internal disorganization over 14 days, reflecting these diffusion limitations. In contrast, spheroids treated with mSiO_2_ maintained size and high circularity throughout the culture period. Furthermore, enhanced metabolic performance was observed in nanohybrid spheroids. ATP levels remained sustained or increased in nanohybrid spheroids, while LOX-1 fluorescence, a marker of hypoxia, remained low. Our observations indicate that the incorporation of mSiO_2_ can help maintain spheroid morphology by facilitating more uniform oxygen and nutrient diffusion, thus minimizing localized hypoxia or necrosis that typically leads to morphological distortion [7,8,9,10].

The functional benefits of nanoparticle incorporation were further evident in adipogenic differentiation. Nanohybrid spheroids displayed more homogeneous and spatially extensive lipid accumulation compared to controls, with larger unilocular droplets reaching deeper into the spheroid core. Gene expression analysis confirmed upregulation of key adipogenic markers, including PPARγ, adiponectin, and FABP4, indicating more robust and functionally mature differentiation. These results support our hypothesis that mSiO_2_ can enhance both structural and functional aspects of 3D adipose cultures. Together, these findings show that the incorporation of mSiO_2_ enhances both the structural stability and the functional differentiation capacity of 3D adipose spheroids, providing a microenvironment that more closely resembles native adipose tissue [7,8,9,10,11,12,13].

Beyond differentiation, the nanohybrid spheroids provided a physiologically relevant platform for studying bacteriophage penetration and distribution in mammalian tissue-like environments. Previous work has shown that bacteriophages can adhere to mammalian cell layers and undergo non-specific internalization, such as through macropinocytosis, without implying infection [33]. Building on this knowledge, our study extends these observations from 2D monolayers to a 3D adipose spheroid model, where diffusion barriers and cell–cell/extracellular matrix interactions more closely mimic in vivo conditions. This 3D environment enabled spatiotemporal mapping of phage penetration, surface association, and uptake [33,34]. This aspect is especially significant because adipose tissue possesses both metabolic and immune functions, yet its role in phage biology has been scarcely examined [35]. By comparing pre-adipose and mature adipocyte spheroids, we revealed that cellular differentiation markedly alters phage behavior, with pre-adipose states permitting rapid and widespread uptake while mature adipocytes displayed delayed, peripheral, and restricted penetration. These results suggest that adipogenic differentiation modifies membrane properties and tissue architecture in ways that significantly influence viral trafficking [33].

The observation that phages accumulate at mammalian cell layers and display reduced penetration in differentiated adipose spheroids has direct implications for phage therapy. Clinical application of phages against drug-resistant infections is limited not only by their short intrabody half-lives but also by host clearance mechanisms, including adherence to and internalization by non-bacterial cells acting as phage sinks [33,35,36,37,38]. Our model highlights adipose tissue as a potential reservoir that may sequester circulating phages and limit their systemic bioavailability. This finding aligns with emerging reports that phages can associate with mammalian epithelia and immune cells, yet extends this knowledge by providing quantitative evidence of how tissue architecture and differentiation state shape these distribution dynamics [33].

Beyond therapeutic considerations, our study underscores the necessity of tissue-mimetic models for evaluating phage pharmacokinetics and biodistribution. While 2D cultures provide useful baseline information, they underestimate the spatial and temporal complexity of phage trafficking. The adipose nanohybrid spheroid system overcomes these limitations and can serve as a physiologically relevant platform for probing tissue-specific phage dynamics. Such insight will be critical for the rational design of tissue-targeted phage delivery strategies, particularly in metabolic or inflammatory disease contexts where adipose tissue plays an active role.

Nevertheless, several limitations should be acknowledged. Our experiments relied on a single phage type and cell source, and thus the observed dynamics may not fully capture the diversity of phage tissue-level distribution in vivo. Additionally, while fluorescence imaging provided valuable penetration depth measurements, complementary approaches such as live imaging in vascularized spheroid systems or in vivo validation will be required to generalize these findings. Future studies could also integrate immune components into the 3D platform to evaluate how macrophages, lymphocytes, or stromal cells influence phage distribution and clearance within adipose tissue.

In summary, this work establishes that both cellular differentiation state and tissue architecture critically determine phage association, penetration, and uptake. Consistent with prior observations in 2D cultures showing that phages can adhere to mammalian cells and undergo non-specific internalization without infection [33], our findings extend this knowledge to a 3D adipose spheroid system.

## 5. Conclusions

In this study, we developed adipose spheroids incorporating mesoporous silica nanoparticles. The nanohybrid spheroids maintained structural integrity for extended culture periods and showed reduced hypoxic stress with higher metabolic activity. Under adipogenic induction, they supported enhanced lipid accumulation and upregulation of adipogenic markers. Using this model, we further observed that phage penetration differed with cell state, as pre-adipocyte spheroids permitted deeper uptake while mature adipocytes restricted phages to peripheral regions. Together these findings confirm that cellular differentiation state critically influence spheroid stability and phage distribution in 3D adipose systems. Beyond these results, the established platform offers practical applications in drug delivery research, tissue engineering, and the study of phage behavior in a physiologically relevant 3D adipose model.

## Data Availability

The original contributions presented in this study are included in the article. Further inquiries can be directed to the corresponding author(s).

## Supplementary Materials

The following supporting information can be downloaded at: https://www.mdpi.com/article/10.3390/nano15191537/s1, Figure S1: Synthesis and Physiochemical characterization of mSiO_2_; Figure S2: Hematoxylin and Eosin (H&E) staining for the analysis of spheroid structural organization; Figure S3: Primer sequences used for RT-PCR analysis of adipogenic markers in 3D hADSC spheroids.
