# Supplementary material for: Development of Silica Nanoparticles Embedded Adipose Spheroid Platform for Probing Bacteriophage Sequestration and Its Implications for Phage Therapy
Source: Nanomaterials (Basel). 2025 Oct 9;15(19):1537. doi: 10.3390/nano15191537 (PMC12526285; doi:10.3390/nano15191537)
Supplement: Supplementary file 1 [file nanomaterials-15-01537-s001.zip › nanomaterials-3882543-supplementary.pdf]

Supporting Information

Development of Silica Nanoparticles Embedded Adipose Spheroid Platform for Probing Bacteriophage Sequestration and Its Implications for Phage Therapy

Rafael Levandowski.[1], Su Yati Htun[1] and Laura Ha[1,]*

1 Department of Pharmaceutical Engineering and Biotechnology, 70 Sun Moon-Ro 221, Tangjeong-Myeon, Asan-Si, 31460, Chungnam, Republic of Korea;

* Correspondence: lauraha1@sunmoon.ac.kr (L.H.); Tel.: + 82-10-4330-2776

Table of Contents

1. **Supporting Figures**

    - **Figure S1.** Synthesis and Physicochemical characterization of $mSiO_2$.
    - **Figure S2. A**nalysis of spheroid structural organization
    - **Figure S3.** Primer sequences used for RT-PCR analysis of adipogenic markers in 3D hADSC spheroids.

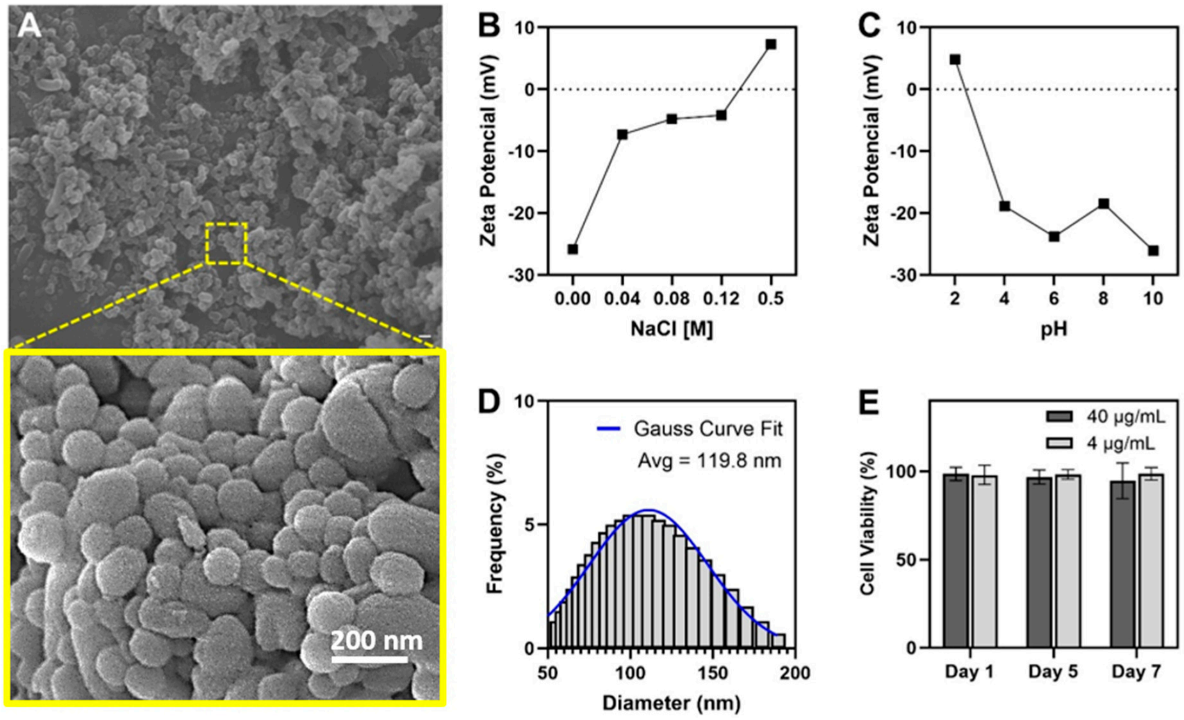

Figure S1. Synthesis and Physicochemical characterization of $mSiO_2$. (A) SEM micrograph showing spherical nanoparticles (scale bar: 200 nm). (B) Zeta potential measured at increasing NaCl concentrations. (C) Zeta potential measured across pH values. (D) Particle-size distribution fitted with a Gaussian curve. (E) Cell viability after exposure to $mSiO_2$ (4 and 40 µg/mL) for 1, 5, and 7 days (n=3/group).

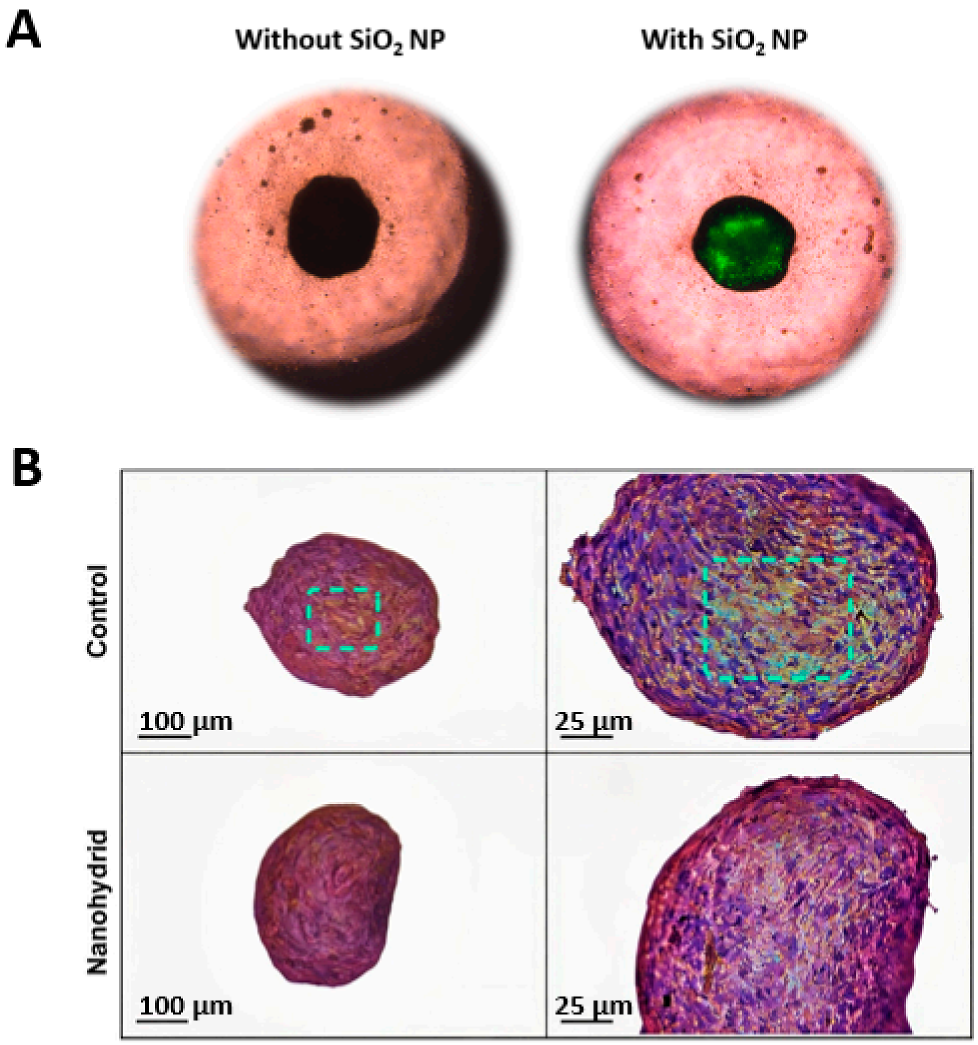

Figure S2. (A) Fluorescence micrographs of 3D spheroids developed with and without FITC-SiO_2 nanoparticles (100ng per spheroid). **(B)**Histological evaluation of spheroid structural organization in control and nanohybrid groups using Hematoxylin and Eosin (H&E) staining.

Gene	Forward Primer (5' → 3')	Reverse Primer (5' → 3')
**FABP4**	ATGGGATGGAAAATCAACCA	GTGGAAGTGACGCCTTTCAT
**PPARγ**	AGATCCAGTGGTTGCAGATT	GGAGATGCAGGCTCCACTTT
**ADIPOQ**	TGGTTTCCAACATGACCTGA	CGAGCTGTGGCCTCATGTA
**18s rRNA**	CGGCTACCACATCCAAGGAA	GCTGGAATTACCGCGGCT

Figure S3. Primer sequences used for RT-PCR analysis of adipogenic markers in 3D hADSC spheroids. Forward and reverse primer sequences targeting FABP4, PPARγ, Adiponectin (ADIPOQ), and 18s ribosomal RNA (housekeeping control) are listed.
